# Supplementary material for: Satellite-based meteorological drought indicator to support food security in Java Island
Source: PLoS One. 2022 Jun 3;17(6):e0260982. doi: 10.1371/journal.pone.0260982 (PMC9165873; doi:10.1371/journal.pone.0260982)
Supplement: S1 File — (ZIP) [file pone.0260982.s001.zip › Supporting Information/Raw and Processed Data.pdf]

Due to file size exceeds the limitation, we ca not upload the dataset with the other supporting information. However, we provide the link that you can access to download the data used in our study.

The raw and processed data are uploaded on the link below:
https://drive.google.com/drive/folders/1qH3xgmk6yPtvpKTqgDFzVNNflRyzUpu2?usp=sharing
