# Supplementary material for: Satellite-based meteorological drought indicator to support food security in Java Island
Source: PLoS One. 2022 Jun 3;17(6):e0260982. doi: 10.1371/journal.pone.0260982 (PMC9165873; doi:10.1371/journal.pone.0260982)

Map Data (Figure 1)

Figure 1 was made from an ESRI ArcGIS base map imagery, and shapefile that was downloaded from OpenStreetMap (https://export.hotosm.org/en/v3/).

Satellite Imagery Data (Figures 2 to 4)

All the satellite imagery data that was used in this study (Figures 2 to 4) were from open-source resources. The authors confirm that the data supporting the findings of this study are available within the following URLs. The URLs provide precipitation data (raw data) where each dataset was downloaded. The SPI data (processed data), authors upload together in this supporting information ZIP file.

SA-OBS : https://sacad.database.bmkg.go.id/
TRMM : https://disc.gsfc.nasa.gov/mirador-guide
PERSIANN : https://chrsdata.eng.uci.edu/
CHIRPS : https://data.chc.ucsb.edu/products/CHIRPS-2.0/

Software License

All the data were processed and analyzed with three different software, NCAR Command Language version 6.6.2, R version 3.6.3, and ArcGIS version 10.6. The NCAR and R are open-source software that can be downloaded with the following URLs.

NCAR : https://www.ncl.ucar.edu/
R : https://www.R-project.org

The ESRI ArcGIS used in this study is licensed for use by National Research Priority (PRN) Program: Drought Model Development for Sustainable Food Estate Management Based on Nexus-Spatial Approach study team in education and research with license reference number ID-IQ2020-11-017.

Data Processing Stages
This flowchart below shows how we processed the data from raw data until we got the SPI Value.

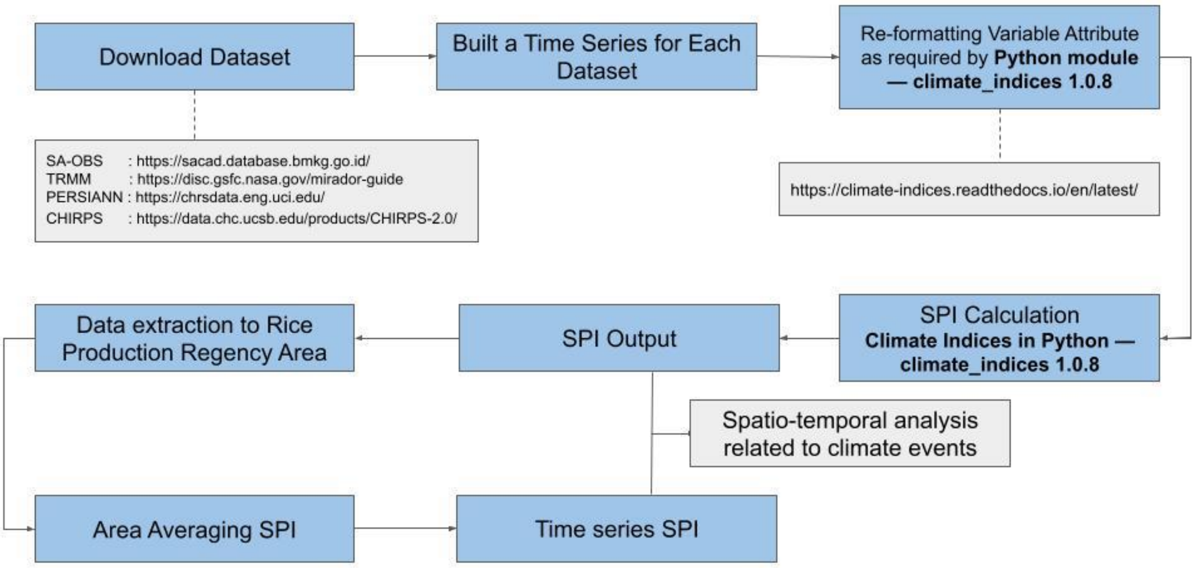

Supplement: S1 File — (ZIP) [file pone.0260982.s001.zip › Supporting Information/Supporting Information.pdf]
