# Supplementary material for: Satellite-based meteorological drought indicator to support food security in Java Island
Source: PLoS One. 2022 Jun 3;17(6):e0260982. doi: 10.1371/journal.pone.0260982 (PMC9165873; doi:10.1371/journal.pone.0260982)
Supplement: Attachment — Submitted filename: PONE-D-21-26705_reviewer.pdf [file pone.0260982.s002.pdf]

PLOS ONE

Meteorological drought indicator based on multisource satellite data and the Standardized Precipitation Index: Application to Java Island in support of food security

--Manuscript Draft--

**Manuscript Number:**	PONE-D-21-26705
**Article Type:**	Research Article
**Full Title:**	Meteorological drought indicator based on multisource satellite data and the Standardized Precipitation Index: Application to Java Island in support of food security
**Short Title:**	Satellite-based meteorological drought indicator to support food security in Java Island
**Corresponding Author:**	Muhammad Dimyati<br>Universitas Indonesia Fakultas Matematika dan Ilmu Pengetahuan Alam<br>Depok, Jawa Barat INDONESIA
**Keywords:**	meteorological; drought; satellite-based precipitation; CHIRPS; TRMM; PERSIANN; SA-OBS; standardized precipitation index; El Niño; IOD; food security; Java Island
**Abstract:**	A meteorological drought refers to reduced rainfall conditions and is a great challenge to food security. Predicting a meteorological drought in advance is important for taking actions in anticipation of its effects, but this can be difficult for areas with limited or sparse ground observation data available. In this study, a meteorological drought indicator was developed that applies the Standardized Precipitation Index (SPI) to satellite-based precipitation products from multiple sources. The developed indicator was then applied to Java Island, which has the largest rice-producing districts of Indonesia. A comparison with ground observation data showed that the satellite products accurately described meteorological drought events in Java both spatially and temporally. Meteorological droughts were found to be strongly dependent on the natural variations in El Niño and a positive-phase Indian Ocean Dipole (IOD). The drought severity was found to be dependent on the intensity of El Niño and a positive-phase IOD that occurs simultaneously, while the duration seems to be modulated more by the positive-phase IOD. The results demonstrate the potential applicability of satellite-based precipitation monitoring to predicting meteorological drought conditions several months in advance and preparing for their effects.
**Order of Authors:**	Siswanto Siswanto
	Kartika Kusuma Wardani
	Babag Purbantoro
	Andry Rustanto
	Faris Zulkarnain
	Evi Anggraheni
	Ratih Dewanti
	Triarko Nurlambang
	Muhammad Dimyati
**Additional Information:**	
**Question**	**Response**
**Financial Disclosure**<br><br>Enter a financial disclosure statement that describes the sources of funding for the work included in this submission. Review the submission guidelines for detailed requirements. View published research	This research was financially supported by National Research Priority (PRN) Ministry of Research and Technology/National Research and Innovation Agency (RISTEK/BRIN) and Indonesia Endowment Fund for Education (LPDP under grant number: 250/E1/PRN/2020.<br>The funders had no role in study design, data collection and analysis, decision to publish, or preparation of the manuscript.

Powered by Editorial Manager® and ProduXion Manager® from Aries Systems Corporation

articles from *PLOS ONE* for specific examples.<br><br>This statement is required for submission and **will appear in the published article** if the submission is accepted. Please make sure it is accurate.<br><br>**Unfunded studies**<br>Enter: *The author(s) received no specific funding for this work.*<br><br>**Funded studies**<br>Enter a statement with the following details:<br>• Initials of the authors who received each award<br>• Grant numbers awarded to each author<br>• The full name of each funder<br>• URL of each funder website<br>• Did the sponsors or funders play any role in the study design, data collection and analysis, decision to publish, or preparation of the manuscript?<br>• **NO** - Include this sentence at the end of your statement: *The funders had no role in study design, data collection and analysis, decision to publish, or preparation of the manuscript.*<br>• **YES** - Specify the role(s) played.<br><br>* typeset	
**Competing Interests**<br><br>Use the instructions below to enter a competing interest statement for this submission. On behalf of all authors, disclose any competing interests that could be perceived to bias this work—acknowledging all financial support and any other relevant financial or non-financial competing interests.<br><br>This statement is required for submission and **will appear in the published article** if the submission is accepted. Please make sure it is accurate and that any funding sources listed in your Funding Information later in the submission form are also declared in your Financial Disclosure statement.	The authors have declared that no competing interests exist.

View published research articles from *PLOS ONE* for specific examples.<br><br>**NO authors have competing interests**<br><br>Enter: *The authors have declared that no competing interests exist.*<br><br>**Authors with competing interests**<br><br>Enter competing interest details beginning with this statement:<br><br>*I have read the journal's policy and the authors of this manuscript have the following competing interests: [insert competing interests here]*<br><br>* typeset	
**Ethics Statement**<br><br>Enter an ethics statement for this submission. This statement is required if the study involved:<br><br>• Human participants<br>• Human specimens or tissue<br>• Vertebrate animals or cephalopods<br>• Vertebrate embryos or tissues<br>• Field research<br><br>Write "N/A" if the submission does not require an ethics statement.<br><br>General guidance is provided below. Consult the submission guidelines for detailed instructions. **Make sure that all information entered here is included in the Methods section of the manuscript.**	N/A

**Format for specific study types**<br><br>**Human Subject Research (involving human participants and/or tissue)**<br>• Give the name of the institutional review board or ethics committee that approved the study<br>• Include the approval number and/or a statement indicating approval of this research<br>• Indicate the form of consent obtained (written/oral) or the reason that consent was not obtained (e.g. the data were analyzed anonymously)<br><br>**Animal Research (involving vertebrate animals, embryos or tissues)**<br>• Provide the name of the Institutional Animal Care and Use Committee (IACUC) or other relevant ethics board that reviewed the study protocol, and indicate whether they approved this research or granted a formal waiver of ethical approval<br>• Include an approval number if one was obtained<br>• If the study involved *non-human primates*, add *additional details* about animal welfare and steps taken to ameliorate suffering<br>• If anesthesia, euthanasia, or any kind of animal sacrifice is part of the study, include briefly which substances and/or methods were applied<br><br>**Field Research**<br><br>Include the following details if this study involves the collection of plant, animal, or other materials from a natural setting:<br>• Field permit number<br>• Name of the institution or relevant body that granted permission	
**Data Availability**<br><br>Authors are required to make all data underlying the findings described fully available, without restriction, and from the time of publication. PLOS allows rare exceptions to address legal and ethical concerns. See the PLOS Data Policy and FAQ for detailed information.	No - some restrictions will apply

Powered by Editorial Manager® and ProduXion Manager® from Aries Systems Corporation

A Data Availability Statement describing where the data can be found is required at submission. Your answers to this question constitute the Data Availability Statement and **will be published in the article**, if accepted.<br><br>**Important:** Stating 'data available on request from the author' is not sufficient. If your data are only available upon request, select 'No' for the first question and explain your exceptional situation in the text box.<br><br>Do the authors confirm that all data underlying the findings described in their manuscript are fully available without restriction?	
**Describe where the data may be found in full sentences. If you are copying our sample text, replace any instances of XXX with the appropriate details.**<br><br>• If the data are **held or will be held in a public repository**, include URLs, accession numbers or DOIs. If this information will only be available after acceptance, indicate this by ticking the box below. For example: *All XXX files are available from the XXX database (accession number(s) XXX, XXX.)*.<br>• If the data are all contained **within the manuscript and/or Supporting Information files**, enter the following: *All relevant data are within the manuscript and its Supporting Information files.*<br>• If neither of these applies but you are able to provide **details of access elsewhere**, with or without limitations, please do so. For example:<br><br>*Data cannot be shared publicly because of [XXX]. Data are available from the XXX Institutional Data Access / Ethics Committee (contact via XXX) for researchers who meet the criteria for access to confidential data.*<br><br>*The data underlying the results presented in the study are available from (include the name of the third party*	One of the data is available from the Indonesian Agency for Meteorology, Climatology, and Geophysics (BMKG). Contact via siswanto.bmkg@gmail.com for researchers who meet the criteria for access to confidential data.

and contact information or URL).<br>• This text is appropriate if the data are owned by a third party and authors do not have permission to share the data.<br><br>* typeset	
Additional data availability information:	Tick here if the URLs/accession numbers/DOIs will be available only after acceptance of the manuscript for publication so that we can ensure their inclusion before publication.; Tick here if your circumstances are not covered by the questions above and you need the journal's help to make your data available.

Powered by Editorial Manager® and ProduXion Manager® from Aries Systems Corporation

Manuscript

Click here to access/download;Manuscript;Manuscript.docx

Satellite-based meteorological drought indicator to support food security in Java Island

Siswanto, Siswanto[1], Kartika Kusuma Wardani[2,3], Babag Purbantoro[4], Andry Rustanto[2], Faris Zulkarnain[2,3], Evi Anggraheni[5], Ratih Dewanti[3], Triarko Nurlambang[2,3,6,7,8], Muhammad Dimyati[2*]

[1] Center for Applied Climate Information and Services, Agency for Meteorology, Climatology, and Geophysics (BMKG), Jakarta, Indonesia

[2] Department of Geography, Faculty of Mathematics and Natural Science, Universitas Indonesia, Depok, Indonesia

[3] Center for Applied Geography Research, Universitas Indonesia, Depok, Indonesia

[4] Remote Sensing Technology and Data Centre, Indonesian Institute of Aeronautics and Space (LAPAN), Jakarta, Indonesia

[5] Department of Civil and Environmental Engineering, Faculty of Engineering, Universitas Indonesia, Depok, Indonesia

[6] Research Center for Climate Change Universitas Indonesia, Depok, Indonesia

[7] Disaster Risk Reduction Center Universitas Indonesia, Depok, Indonesia

[8] Sustainable Development Goals Hub Universitas Indonesia, Depok, Indonesia

*Corresponding author

Email: m.dimyati@sci.ui.ac.id (MD)

The abstract and the entire text are not well-aligned, this need to be addressed.

Abstract

A meteorological drought refers to reduced rainfall conditions and is a great challenge to food security. Predicting a meteorological drought in advance is important for taking actions in anticipation of its effects, but this can be difficult for areas with limited or sparse ground observation data available. In this study, a meteorological drought indicator was

How did you developed met. drought?,

developed that applies the Standardized Precipitation Index (SPI) to satellite-based precipitation products from multiple sources. The developed indicator was then applied to Java Island, which has the largest rice-producing districts of Indonesia. A comparison with ground observation data showed that the satellite products accurately described meteorological drought events in Java both spatially and temporally. Meteorological droughts were found to be strongly dependent on the natural variations in El Niño and a positive-phase Indian Ocean Dipole (IOD). The drought severity was found to be dependent on the intensity of El Niño and a positive-phase IOD that occurs simultaneously, while the duration seems to be modulated more by the positive-phase IOD. The results demonstrate the potential applicability of satellite-based precipitation monitoring to predicting meteorological drought conditions several months in advance and preparing for their effects.

How did you confirm this?

Keywords: meteorological, drought, satellite-based precipitation, CHIRPS, TRMM, PERSIANN, SA-OBS, standardized precipitation index, El Niño, IOD, food security, Java Island

Too many keywords, six keywords should suffice, rearrange the keywords alphabetically

Introduction

In Indonesia, various regions have reported drought events indicated by a scarcity of surface water [1,2], which may be followed by depletion of the shallow groundwater table [3] and reduced crop productivity [4,5]. These events are mainly induced by the high seasonality of the rainfall, which is particularly erratic for regions located farthest south from the Equator such as Java, Nusa Tenggara, and the southern parts of Sumatra, Kalimantan, and Papua [6,7]. Drought events in Indonesia are closely related to global climate phenomena, particularly El Niño [6,8] and the Indian Ocean Dipole (IOD) [8]. A strong El Niño may induce intense fires in forest and peatland areas [9] that cause transboundary haze or smoke. There is a need for sustained monitoring and accurate forecasting of meteorological conditions to anticipate and minimize the negative effects of drought events. Droughts can be classified as meteorological, agricultural, hydrological, or socioeconomic. At present, Indonesia has no services that provide comprehensive drought information to the public. The Indonesian Agency for Meteorology, Climatology, and Geophysics (BMKG) only provides a meteorological drought warning service.

A meteorological drought refers to a temporary reduction in the amount of precipitation compared to the average [10,11]. The degree of the drought is determined according to the ratio between the amount of precipitation and the amount considered normal for that period, and the duration is determined by how long the dry condition persists. A meteorological drought affects the agricultural sector by causing a groundwater deficit, which can result in crop failure, and reducing the water reserves of dams available for irrigation. If a meteorological drought persists, a hydrological drought may occur, which is defined as a reduced supply of surface water and groundwater and is based on the measured water levels of rivers, reservoirs, lakes, and groundwater [12]. BMKG defines a meteorological drought as an area experiencing dry conditions for a certain time due to reduced precipitation or a longer

More recent citations should be included, see few below;
https://doi.org/10.1016/j.heliyon.2019.e02148
https://iopscience.iop.org/article/10.1088/1755-1315/648/1/012130/meta
https://doi.org/10.1007/s11069-020-04421-x

dry season than normal, and it issues warnings based on the number of consecutive days without rain (i.e., dry spell) and prediction of a low precipitation amount. Predicting dry spells is very important because they have a major influence on agriculture, especially rice production [13,14]. Prolonged dry spells can reduce the yield [15] and even the overall rice production [16]. Dry spells are the most sensitive indicator of the effect of El Niño on Indonesia [17].

BMKG monitors for meteorological droughts on a 10-day timescale, which is complemented by near-real-time monitoring of the daily precipitation product estimated from Global Satellite Mapping of Precipitation (GSMaP) data. However, the utilization of available multisource or selected satellite precipitation products is an ongoing challenge that needs to be addressed to improve the comprehensiveness, speed, and accuracy of drought information services in the spatial and temporal domains. Using satellite products for near- and post-real-time precipitation estimation can greatly improve the quality of drought analysis. Several high-resolution precipitation products derived from meteorological satellites with worldwide coverage have recently become freely available in near real time with a horizontal resolution of 0.05°–0.25° [18–21]. These include Tropical Rainfall Measuring Mission (TRMM) and its successor Global Precipitation Measurement (GPM), GSMaP, Climate Hazards Group InfraRed Precipitation with Stations (CHIRPS), and Precipitation Estimation from Remotely Sensed Information using Artificial Neural Networks–Climate Data Record (PERSIANN-CDR).

Several studies have evaluated satellite products for the best fit to ground observations of precipitation and meteorological drought. CHIRPS showed the closest agreement to rain gauge data compared with PERSIANN-CDR and TRMM for the Bundelkhand region of central India [22]. PERSIANN-CDR and CHIRPS were found to be more accurate than TRMM for meteorological drought monitoring of Chile in South America [23]. PERSIANN-

CDR performed better than CHIRPS for semiarid regions of Brazil [24]. GPM, GSMaP, and PERSIANN were all found to offer reliable near-real-time meteorological estimation of data-sparse regions in Iran [25]. These satellite products have demonstrated an acceptable accuracy compared to rain gauge data in some areas of Indonesia, although there have been some difficulties and differences, particularly in the representation of extremely heavy rain conditions [26–29]. CHIRPS was found to correlate well with rain gauge data from six meteorological stations installed by BMKG with a correlation coefficient of 0.70–0.86 [30]. CHIRPS outperformed GSMaP and IMERG at assessing the monthly precipitation of Bali Island [29]. TRMM performed better than Asian Precipitation–Highly-Resolved Observational Data Integration Towards Evaluation (APHRODITE) for the Pemali–Comal River basin [31].

In this study, we developed a meteorological drought indicator that applies the widely used Standardized Precipitation Index (SPI) to precipitation products from multiple satellite data sources. This differs from currently available where the SPI is calculated based on sparse surface observation data. The evaluation of multi-source SPI with El Niño and IOD simultaneously is expected to provide a better, optimal, and more accurate representation and understanding of meteorological drought events in the study area. We used the proposed indicator to evaluate the satellite precipitation products against ground observation data for Java Island, with a focus on eight of the 10 largest rice-producing districts of Indonesia: Karawang, Subang, Indramayu, Cilacap, Grobogan, Sragen, Ngawi, and Lamongan (Fig 1). These districts have a very low density of rain gauges and other surface meteorological observation networks. Accurate forecasting of meteorological drought conditions for these districts is fundamental for food security by allowing countermeasures to be performed early in advance. This study may also help address the effects of climate change on Java, which has

experienced a continuous decrease in precipitation during the dry season over the last three decades (1981–2010) [32].

What is the identified gap that this study is filling? this is not clearly stated, address this during revision

Fig 1. Study area: Java Island and its position within the Indonesian archipelago. The colored polygons indicate the eight districts as the largest rice producer in Indonesia.

Data and methods

Data

We used monthly precipitation estimated from four datasets covering Java with similar spatial resolutions. Three datasets were satellite-based precipitation products: CHIRPS, TRMM, and PERSIANN. The Southeast Asia Observational Dataset (SA-OBS) was used to validate the satellite products. Table 1 summarizes the datasets, their resolutions, and time covered. The datasets are briefly described below.

Table 1. Summary of the Datasets Used in this Study.

Data source	Temporal resolution	Spatial resolution	Time period
CHIRPS	Monthly	0.05° × 0.05°	January 1981–January 2021
TRMM	Monthly	0.25° × 0.25°	January 1998–January 2020
PERSIANN	Monthly	0.25° × 0.25°	March 2000–April 2021
SA-OBS	Monthly	0.25° × 0.25°	January 1981–December 2017

Climate Hazards Group Infrared Precipitation with Stations

CHIRPS is a satellite product with multiple data sources [33] that is available at high resolution (0.05° × 0.05°) and a long time series from 1981 to the present [34]. Regional evaluations have shown that estimations using CHIRPS agree well with ground observations from local gauge networks, which is promising for drought monitoring [35]. In this study, we used the CHIRPS v.2 Global Monthly dataset for the period March 1981–January 2021.

Tropical Rainfall Measuring Mission

TRMM has three types of data products: orbital (also known as swath), gridded, and TRMM-related (e.g., ancillary products, ground-based instrument products, TRMM and ground observation subsets, and field experiment products) [36]. In this study, we used a gridded data product: the TRMM (TMPA) 3B43 V7 Monthly dataset for the period March 1998–January 2020. TMPA 3B43 is a widely used dataset because of its high spatial and temporal resolutions. The latest version (Version 7) includes a uniform data reprocessing and calibration scheme and a single use of Global Precipitation Climatology Centre monthly rain gauge analysis, which improves the accuracy compared to previous versions [28].

Precipitation Estimation from Remotely Sensed Information using Artificial Neural Networks

PERSIANN is based on geostationary infrared imagery and daytime visible imagery. This system was first developed in 2000, and it estimates surface precipitation rates based on local cloud texture characteristics by using infrared images with an artificial neural network (ANN) [37,38]. The ANN generates global precipitation for the latitudes of 60° S to 60 °N in two steps: an automatic clustering process is applied to transforming infrared (wavelength of 10.2–11.2 μm) images into a hidden layer to form a self-organizing feature map (SOFM); then the discrete SOFM clusters in the hidden layer are made continuous [39,40].

Southeast Asia Observational Dataset

SA-OBS is a newly available high-resolution land-only gridded dataset for Southeast Asia. It provides the daily precipitation amount and daily minimum, mean, and maximum temperatures. It was developed by BMKG and the Royal Netherlands Meteorological Institute (KNMI) with the aim of providing an observational basis against which model results can be compared [41]. SA-OBS can be compared against existing gridded datasets based either on a less dense network of stations covering the same area or on satellite data where the

interpretation of infrared and microwave signals as precipitation or temperature has limited accuracy, such as APHRODITE [42,43], CMORPH [44], and TRMM [45]. We used a SA-OBS dataset with a regular 0.25° × 0.25° grid resolution for the period of January 1981–December 2017.

We also used monthly data of the Oceanic Nino Index (ONI) and Dipole Mode Index (DMI) to monitor the activities of the El Niño–Southern Oscillation (ENSO) in the Pacific Ocean and the IOD in the Indian Ocean. The ONI was obtained from the National Weather Service Climate Prediction Center (https://origin.cpc.ncep.noaa.gov/), while the DMI was retrieved from the Global Climate Observing System (https://psl.noaa.gov/).

Methods

We used the SPI to determine meteorological drought conditions in the study area. The SPI is commonly used for meteorological drought analysis and can be calculated for various time scales to evaluate not only the water supply in the short term but also the available water resources in the long term [46,47]. McKee et al. developed the SPI in 1993 based on their understanding that a precipitation deficit has different effects on the groundwater, reservoir storage, soil moisture, snowpack, and streamflow [10,48]. The SPI is computed by measuring precipitation anomalies at a given location, which are identified by comparing the observed total precipitation with the long-term historical record for a period of interest (e.g., 1, 3, 12, or 49 months) [49]. The historical record is fitted to a gamma probability distribution, which is then transformed into a normal distribution such that the mean SPI for the given location and period is zero. The SPI is calculated as follows [46]:

$$x_s^m(t) = \sum_{i=g-s+1}^{g} x(i),\ g = 12(t-1) + m \qquad (1)$$

where *x* is the monthly precipitation series, *t* is the yearly index, and *m* is a specific month (e.g., January, February).

For a given location, a rainfall deficit (i.e., meteorological drought) occurs when the SPI is less than –1.0, while excess rainfall occurs when the SPI is greater than 1.0 (Table 2). Because the SPI is given in units of standard deviation from the long-term mean, it can be used to compare precipitation anomalies for any geographic location and any number of timescales. In this study, we used the 3-month (SPI-3) and 6-month (SPI-6) timescales. SPI-3 compares the precipitation over a specific 3-month period with the precipitation over the same 3-month period for all years included in the dataset; it reflects short- and medium-term moisture conditions and provides a seasonal estimation of precipitation, which is useful for evaluating the available water supply for primary agricultural regions [50]. Similarly, SPI-6 compares the precipitation over a specific 6-month period with the precipitation over the same 6-month period for all years included in the dataset. SPI-6 indicates seasonal to medium-term trends in precipitation and may also be associated with anomalous streamflows and reservoir levels depending on the location and time of year [50].

In this study, we calculated the SPI by using the open-source module Climate and Drought Indices in Python [51]. All analysis and plots were performed with NCAR Command Language (https://www.ncl.ucar.edu), R (https://www.R-project.org), and ArcGIS (https://www.esri.com).

Table 2. Drought Severity based on SPI values [50].

2.0+	Extremely wet
1.5 to 1.99	Very wet
1.0 to 1.50	Moderately wet
−0.99 to 0.99	Near normal
−1.0 to −1.50	Moderately dry
−1.5 to −1.99	Severely dry
−2 and less	Extremely dry

Figures are not of high quality, clearer figures should be considered.

Results

Accuracy of the satellite products at describing precipitation climatology

Fig 2 shows the annual total precipitation (left), mean monthly precipitation during the peak of the rainy season (middle), and mean monthly precipitation during the dry season (right) of Java according to the satellite products (CHIRPS, TRMM, PERSIANN) and observational dataset (SA-OBS) for 2001–2020. We analyzed the original datasets without ocean masking or changing the horizontal resolution. The three satellite products returned similar and comparable results for the total annual precipitation that agreed with SA-OBS; in general, West Java was wetter than other parts of the study area. CHIRPS and TRMM accurately described the parts of the study area with the highest precipitation intensity (>3200 mm/year): the southern part of West Java and central part of Central Java. However, PERSIANN did not return such a high intensity. These parts of the study area with a high annual precipitation are dominated by highlands or mountains. PERSIANN also showed a wider distribution of precipitation over the study area but failed to describe the parts with the highest precipitation. TRMM also identified a different location with the highest precipitation: the southern part of West Java. In general, the precipitation tended towards areas close to the sea. Seven of the rice-producing districts had an annual precipitation of 2000–2510 mm/year, while Cilacap received even more. The variation in annual precipitation is generally determined by the precipitation variations at the peaks of the rainy and dry seasons. The peak of the rainy season is defined as the months with the highest precipitation intensity and frequency. In contrast, the peak of the dry season is defined as the months with the smallest reduction in the precipitation amount and frequency. For Java, the peaks of the rainy and dry seasons are typically December–February (DJF) and June–August (JJA), respectively.

During the peak of the rainy season, almost all of the datasets indicated that the central mountainous part of Central Java had the highest precipitation intensity in the study area. PERSIANN slightly differed by indicating a wider distribution area. CHIRPS, TRMM, and SA-OBS showed good agreement on the precipitation distribution for the study area, with particularly strong agreement between CHIRPS and SA-OBS. During this period, the eight rice-producing districts generally received about 251–400 mm/month of precipitation.

At the peak of the dry season, the precipitation intensity for the entire study area was generally 0–151 mm/month. All datasets indicated that West Java was wetter and East Java was drier with varying spatial distributions. PERSIANN and SA-OBS agreed on the dry conditions of East Java, while CHIRPS and TRMM indicated slightly wetter conditions. CHIRPS, TRMM, and SA-OBS agreed on the wetter conditions of West Java. All rice-producing districts in West Java and Cilacap received monthly precipitation of about 100 mm/month, while rice-producing districts in eastern Central Java and East Java experienced very low monthly precipitation of <51 mm/month.

The peak of the rainy season corresponds to December, January, and February (DJF); the peak of the dry season corresponds to June, July, and August (JJA).

Fig 2. Comparison of the annual total precipitation (left), mean monthly precipitation in the rainy season (middle), and mean monthly precipitation in the dry season (right) over the study area according to 20 years of climatological data from CHIRPS, TRMM, PERSIANN, and SA-OBS. The rainy season corresponds to December, January, and February (DJF); the dry season corresponds to June, July, and August (JJA). Dots on the map indicate the largest rice-producing districts of Indonesia.

Accuracy of the satellite products at describing drought events: spatial and temporal domains

In Java, drought conditions are generally influenced by El Niño and a positive-phase IOD. Drought events strongly affect agricultural productivity in Java. We needed to determine the reliability of satellite-based precipitation products at describing the spatial and temporal distributions of drought events. As an example, we applied the SPI to describe the spatial distribution of drought conditions in Java for a drought event that occurred in mid-2015 that was caused by a strong El Niño in the Pacific Ocean and positive-phase IOD in the Indian Ocean. Fig 3 shows the SPI-3 results for the spatial distribution of the meteorological drought event in June (left), August (middle), and October (right). All datasets showed that the drought started from the western part of Java in June. CHIRPS and SA-OBS agreed that the drought was uniformly distributed in West Java, but TRMM and PERSIANN indicated that the drought was more concentrated on the north shore. SA-OBS had high absolute values for SPI-3 of less than −2.4 in some parts of the study area while the surrounding parts had SPI values of more than −1.2. SA-OBS had blank SPI-3 values for some parts of the study area, which was attributed to a possible lack of observations for that time. No significant drought was detected in Central Java and East Java in June. The largest rice-producing districts in West Java and the western part of Central Java were affected by meteorological drought conditions based on the SPI being between −1.2 and −2.0 (moderately to very dry). All datasets showed a gradual spatial expansion of intense meteorological drought conditions toward the east of the study area in August and October. The SPI values of TRMM, PERSIANN, and SA-OBS indicated that very dry conditions had spread over almost all of Java by August, including the eight largest rice-producing districts, and extremely dry conditions (below −2) had spread in some parts of West Java by October. Because of its

higher spatial resolution, CHIRPS gave a smoother distribution than the other datasets. SA-OBS returned a rougher distribution in some areas because of the sparse observational data.

Fig 3. SPI-3 derived from CHIRPS, TRMM, PERSIANN, and SA-OBS for June (left), August (middle), and October 2015 (right) during the drought event due to the 2015–16 El Niño. Dots on the map indicate the largest rice-producing districts of Indonesia.

Similar to Fig 3, Fig 4 presents the spatial distribution of SPI-6 values over Java for June (left), August (middle), and October 2015 (right). Compared to CHIRPS and TRMM, SA-OBS and PERSIANN indicated drier conditions in June, while all datasets indicated a comparable expansion of drought conditions in August. From June, SA-OBS indicated moderate to very dry conditions (SPI-6 of −1 to −2) over the western half of Java and extremely dry conditions (SPI-6 of less than −2) in Central Java. All datasets indicated that the meteorological drought had worsened over all of Java by October. Moreover, most of the eight largest rice-producing districts were suffering from the meteorological drought with very dry to extremely dry conditions. The occurrence of an El Niño appears to trigger anomalies in the river flow and reservoir levels of the study area 5–6 months later; this can be used as an early warning for the agricultural sector to take action against potential drought events.

Fig 4. Six-month SPI (SPI-6) derived from CHIRPS, TRMM, PERSIANN, and SA-OBS for June 2015 (left), August 2015 (middle), and October 2015 (right) during the drought event due to the 2015–16 El Niño. Dots on the map indicate the largest rice-producing districts of Indonesia.

The reliability of the satellite products also needed to be evaluated in the temporal domain to determine the suitability of the SPI as a tool for drought monitoring. We focused on the drought characteristics for the eight largest rice-producing districts of Java: Karawang, Subang, and Indramayu in West Java Province; Cilacap, Grobogran, and Sragen in Central

Java Province; and Ngawi and Lamongan in East Java Province. Fig 5 depicts the time series of the monthly precipitation (left) and SPI-3 (right) of these districts grouped by province: West Java (top), Central Java (middle), and East Java (bottom). The monthly precipitation and SPI-3 were derived from CHIRPS (blue line), TRMM (green line), PERSIANN (red line), and SA-OBS (black line). The time series were obtained for all available periods covered by the datasets. In general, the four datasets showed similar evolutions and variations in precipitation and drought conditions for all locations. Differences were observed in the monthly precipitation intensity; for example, TRMM or PERSIANN sometimes indicated a higher or lower monthly precipitation intensity than the other datasets. Consequently, SPI-3 values from TRMM and PERSIANN sometimes indicated drier or wetter conditions than the other datasets. For certain locations (e.g., West Java and Central Java), PERSIANN sometimes exhibited large deviations or even the opposite trend from the other datasets, which may be due to its algorithms.

Fig 5. Time series for the monthly precipitation (left) and SPI-3 (right) of the eight largest rice-producing districts in Java grouped by province: West Java (top), Central Java (middle), and East Java (bottom). The monthly precipitation and SPI-3 were determined from CHIRPS (blue), TRMM (green), PERSIANN (red), and SA-OBS (black).

For a quantitative view, Fig 6 evaluates the accuracy of the monthly precipitation and SPI-3 derived from satellite products in terms of their proximity to the observational data, which is indicated by the correlation R. We assumed that the SA-OBS dataset is the ground truth and validated the satellite products against them. The satellite products were concluded to be valid if the data distribution was centered or converged to the 1:1 reference line, and the slope of the regression line closely fit the reference line. We only used the period of 2001–2017 so that all datasets could be compared on the same basis. CHIRPS and TRMM showed higher correlations with SA-OBS than PERSIANN with R values of 0.82 for the monthly

precipitation and >0.6 for SPI-3. For a longer time window (1998–2017), CHIRPS had a higher correlation to SA-OBS than TRMM (not shown).

Fig 6. Scatterplot of the regional pooled precipitation (left) and SPI-3 (right) for 2001–2017. CHIRPS, TRMM, and PERSIANN were each plotted against SA-OBS.

Meteorological drought characteristics in the study area

As noted previously, drought in Indonesia has traditionally been related to global climate phenomena, particularly the ENSO in the Pacific Ocean. However, research on the relation of drought to the IOD is still limited [8]. Fig 7 presents a parallel time series of the ONI and DMI showing the significance of the ENSO and IOD, respectively, as inter-annual climate drivers of the precipitation variability in Indonesia. We wanted to determine if a temporal relationship exists between variations in monthly precipitation and SPI-3 and these phenomena that can be used as an indicator of meteorological drought. We built a multisource averaged SPI from individual SPIs derived from CHIRPS, TRMM, PERSIANN, and SA-OBS. Most of the individual SPIs showed good agreement, except for those from PERSIANN for the last part of the time series. The multisource averaged SPI showed that severe meteorological droughts always occurred in conjunction with a strong El Niño or positive-phase IOD. Strong El Niño events (ONI > +2) occurred in 1982–83, 1997–98, and 2015–16. Other El Niño events were weak or moderate. These strong El Niño events were always followed by meteorological drought events that could be extreme. The peaks of these extreme droughts occur simultaneously with the peaks of ONI with no time lag. The seasonal precipitation patterns persisted even though the intensity was lower than usual during these events. Meteorological droughts with very dry conditions also occurred in 1987, 1992, 1994–95, 2007, and 2015 following weak to moderate El Niño events. The meteorological droughts in response to weak and moderate El Niño events did not occur simultaneously, and the

droughts were moderately to very dry. In 1992, the very dry condition showed a stronger correlation with the IOD timeline rather than El Niño, which peaked later.

For the SPI results, the colored bar shows the average from multiple sources, and the lines show the values derived from individual datasets: CHIRPS (blue), TRMM (green), PERSIANN (red), and SA-OBS (black). For each time series, the vertical orange solid line indicates a year with El Niño, and a vertical light-blue line indicates a year with La Niña. The horizontal gray dashed lines indicate the levels of ENSO (top), precipitation (middle), and SPI (bottom).

Fig 7. Time series of the Oceanic Nino Index (ONI) and Dipole Mode Index (DMI) for climate events during 1981–2020 (top), the monthly precipitation derived from all datasets (middle), and SPIs (bottom). For the SPI results, the colored bar shows the average from multiple sources, and the lines show the values derived from individual datasets: CHIRPS (blue), TRMM (green), PERSIANN (red), and SA-OBS (black). For each time series, the vertical orange solid line indicates a year with El Niño, and a vertical light-blue line indicates a year with La Niña. The horizontal gray dashed lines indicate the levels of ENSO (top), precipitation (middle), and SPI (bottom).

Interestingly, the 2015 drought event only reached very dry conditions even though a strong El Niño took place at the time. The positive-phase IOD was not very strong for this event, so the meteorological drought did not reach extremely dry conditions as in the cases of the El Niño events in 1982 and 1997. On the other hand, an extreme meteorological drought occurred in 2019, even though the El Niño had returned to neutral and was previously weak.

The severe 2019 drought showed a stronger correlation with the positive-phase IOD, which was the strongest event according to historical records. Based on these results, we concluded that the drought severity depends on the intensity of El Niño and positive-phase IOD occurring simultaneously, while the time phase seems to be modulated by the positive-phase IOD.

You failed to compared your result with recent and relevant studies, see few suggestions below;
https://doi.org/10.1016/j.jenvman.2021.112028
https://doi.org/10.1016/j.jenvman.2021.112112
https://doi.org/10.1007/s10661-020-08730-3
https://doi.org/10.1080/19376812.2020.1841658

Discussion

In this study, we showed that the SPI-based meteorological drought indicator derived from satellite products may be applied to drought monitoring with more detail, wider coverage, and a faster timescale than ground observations. The multi-sources SPI-based meteorological drought indicator described well the relation of meteorological drought characteristics in Java island with El Niño and positive-phase IOD. This adds to a better understanding of the role of the Indian Ocean on drought variability in the study area. Several previous studies have characterized the Java Island drought which is influenced by El Niño in the Pacific Ocean [7, 8]. If the SPI or other meteorological drought indicators can be used to predict drought conditions in the next 3 months, then they can be used by the agricultural sector to anticipate and prepare for drought events in the near future. The satellite-based precipitation products can be utilized to predict future conditions including patterns, trends, and seasonal influences for water resource management at different timescales (Murat et al., 2018). This may be very useful for farmers, who need to make decisions on their planting strategies while accounting for the possibility of meteorological droughts that can progress to agricultural droughts. This is also important for water management authorities when estimating the water supply and planning a distribution strategy. The proposed satellite-based meteorological drought indicator can help farmers secure their economic livelihood and help the government guarantee food security. For example, rice production can be maintained by

Check you referencing style

improved water management from several reservoirs and cloud seeding in anticipation of predicted meteorological drought events.

Conclusion

No citation is expected in the conclusion, this is your findings and should be solely yours, this section need to be improved, future research statement should be stated.

We developed an SPI-based meteorological drought indicator, which we applied to multiple sources of satellite precipitation products for the largest rice-producing districts of Java, which are poorly served by ground observations. The results showed that satellite products can be used to accurately describe the spatial and temporal distributions of meteorological drought events in the study area. A comparison with the ground observation dataset (SA-OBS) showed that the CHIRPS and TRMM had better correlations than PERSIANN of up to 0.6. Meteorological drought characteristics in the study area were shown to be strongly dependent on the variations in El Niño and the positive-phase IOD. The drought severity depends on the intensities of El Niño and positive-phase IOD occurring simultaneously, while the time phase seems to be modulated by the positive-phase IOD. Our results indicate the potential applicability of satellite-based precipitation monitoring to predicting meteorological drought events several months in advance, something valuable for an early warning. Further research is still needed to improve the satellite products accuracy by bias correction implementation [7], as well as investigating the time lag of meteorological drought which will have an impact on causing agricultural drought in the study area. If this is provided, it will be very useful as a decision-making tool for farmers and the government to take action early.

The SPI can also be used to indicate wetness in the study area, which seems to be influenced by inter-annual climate variability in the Pacific Ocean and Indian Ocean (i.e., La Niña and negative-phase IOD), although these appear to have different temporal characteristics compared to meteorological droughts. Another study is needed to discuss this topic as it is outside the scope of the present study.

Acknowledgments

We thank the Ministry of Research and Technology/National Research and Innovation Agency (RISTEK/BRIN), The Agency for Meteorology, Climatology and Geophysics (BMKG), and National Institute of Aeronautics and Space (LAPAN), NASA's Earth Data, the Center for Hydrometeorology and Remote Sensing (CHRS), and Climate Hazards Center UCSB for providing data and information for this research.

References

1. Yasa IW, Bisri M, Sholichin M, Andawayanti U. Hydrological drought index based on reservoir capacity - Case study of Batujai dam in Lombok Island, West Nusa Tenggara, Indonesia. J Water L Dev. 2018;38: 155-162.
2. Pramono IB, Savitri E. Modification Method of Drought Vulnerability at Wonogiri District, Central Java, Indonesia. In: Int J Innov Technol Explor Eng [Internet]. 2019;8: 551-555. Available from: https://www.ijitee.org/wp-content/uploads/papers/v8i6s3/F11080496S319.pdf.
3. Taufik M, Setiawan BI, van Lanen HAJ. Modification of a fire drought index for tropical wetland ecosystems by including water table depth. Agric For Meteorol. 2015;203: 1-10.
4. Moser G, Leuschner C, Hertel D, Hölscher D, Köhler M, Leitner D, et al. Response of cocoa trees (Theobroma cacao) to a 13-month desiccation period in Sulawesi, Indonesia. Agrofor Syst. 2010;79: 171-187.
5. Khanal AR, Regmi M. Financial constraints and production efficiency: A case from rice growers in drought prone areas of Indonesia. Agric Financ Rev. 2018;78: 25-40.

6. Aldrian E, Dwi Susanto R. Identification of three dominant rainfall regions within Indonesia and their relationship to sea surface temperature. Int J Climatol. 2003;23: 1435-1462.

7. Vernimmen RRE, Hooijer A, Mamenun, Aldrian E, Van Dijk AIJM. Evaluation and bias correction of satellite rainfall data for drought monitoring in Indonesia. Hydrol Earth Syst Sci. 2012;16: 133-147.

8. D'Arrigo R, Wilson R, Palmer J, Krusic P, Curtis A, Sakulich J, et al. Monsoon drought over Java, Indonesia, during the past two centuries. Geophys Res Lett. 2006;33: 4-7.

9. Taufik M, Setiawan BI, Van Lanen HAJ. Increased fire hazard in human-modified wetlands in Southeast Asia. Ambio. 2019;49: 363-373.

10. McKee TB, Nolan J, Kleist J. The relationship of drought frequency and duration to time scales. Prepr Eighth Conf Appl Climatol Amer Meteor, Soc. 1993; 179-184.

11. Eriyagama N, Smakhtin V, Gamage N. Mapping Drought Patterns and Impacts: A Global Perspective [Internet]. Books.Google.Com. Colombo, Sri Lanka: International Water Management Institute; 2009. 36 p. Available from: http://books.google.com/books?hl=en&lr=&id=kbMxhbIseQAC&oi=fnd&pg=PR6&dq=Mapping+Drought+Patterns+and+Impacts:+A+Global+Perspective&ots=HPelU9jrER&sig=zRBN-21Snyc0ziHsVCbiN7w9rKI.

12. Dai A. Drought under global warming: A review. Wiley Interdiscip Rev Clim Chang. 2011;2: 46-65.

13. Nieuwolt S. Estimating the agricultural risks of tropical rainfall. Agric For Meteorol. 1989;46: 251-263.

14. Zhu X, Troy TJ. Agriculturally relevant climate extremes and their trends in the world's major growing regions. Earth's Futur. 2018;6: 656-672.

15. Wopereis MCS, Kropff MJ, Maligaya AR, Tuong TP. Drought-stress responses of two lowland rice cultivars to soil water status. F Crop Res. 1996;47: 21-39.

16. Castillo EG, Buresh RJ, Ingram KT. Lowland rice yield as affected by timing of water deficit and nitrogen fertilization. Agron J. 1992;84: 152-159.

17. Supari, Tangang F, Salimun E, Aldrian E, Sopaheluwakan A, Juneng L. ENSO modulation of seasonal rainfall and extremes in Indonesia. Clim Dyn. 2018;51: 2559-2580.

18. Kawanishi T, Kuroiwa H, Kojima M, Oikawa K, Kozu T, Kumagai H, et al. TRMM precipitation radar. Adv Sp Res. 2000;25: 969-972.

19. Okamoto K, Ushio T, Iguchi T, Takahashi N, Iwanami K. The global satellite mapping of precipitation (GSMaP) project. In: International Geoscience and Remote Sensing Symposium (IGARSS). 2005.

20. Hou AY, Kakar RK, Neeck S, Azarbarzin AA, Kummerow CD, Kojima M, et al. The global precipitation measurement mission. Bull Am Meteorol Soc. 2014;95: 701-722.

21. Funk C, Peterson P, Landsfeld M, Pedreros D, Verdin J, Shukla S, et al. The climate hazards infrared precipitation with stations - A new environmental record for monitoring extremes. Sci Data. 2015;2: 151066.

22. Pandey V, Srivastava PK, Mall RK, Munoz-Arriola F, Han D. Multi-satellite precipitation products for meteorological drought assessment and forecasting in Central India. Geocarto Int. 2020: 1-20.

23. Zambrano F, Wardlow B, Tadesse T, Lillo-Saavedra M, Lagos O. Evaluating satellite-derived long-term historical precipitation datasets for drought monitoring in Chile. Atmos Res. 2017;186: 26-42.

24. Brito CS de, Silva RM da, Santos CAG, Brasil Neto RM, Coelho VHR. Monitoring meteorological drought in a semiarid region using two long-term satellite-estimated

rainfall datasets: A case study of the Piranhas River basin, northeastern Brazil. Atmos Res. 2021;251.

25. Yazdandoost F, Moradian S, Izadi A, Bavani AM. A framework for developing a spatial high-resolution daily precipitation dataset over a data-sparse region. Heliyon. 2020;6: e05191.

26. As-syakur AR, Tanaka T, Osawa T, Mahendra MS. Indonesian rainfall variability observation using TRMM multi-satellite data. Int J Remote Sens. 2013;34.

27. Sugiartha N, Ogawara K, Tanaka T, Mahendra MS. Application of GSMaP product and rain gauge data for monitoring rainfall condition of flood events in Indonesia. Int J Environ Geosci. 2017;1.

28. Duan Z, Bastiaanssen WGM, Liu J. Monthly and annual validation of TRMM multi satellite precipitation analysis (TMPA) products in the Caspian Sea Region for the period 1999-2003. In: International Geoscience and Remote Sensing Symposium (IGARSS). 2012.

29. Liu CY, Aryastana P, Liu GR, Huang WR. Assessment of satellite precipitation product estimates over Bali Island. Atmos Res. 2020;245.

30. Nugroho JT, Nurfitriani D, Suwarsono, Chulafak GA, Manalu RJ, Harini S. Rainfall anomalies assessment during drought episodes of 2015 in Indonesia using CHIRPS Data. IOP Conf Ser Earth Environ Sci. 2021;739.

31. Levina, Hatmoko W, Seizarwati W, Vernimmen R. Comparison of TRMM satellite rainfall and APHRODITE for drought analysis in the Pemali-comal River Basin. Procedia Environ Sci. 2016;33: 187-195.

32. Siswanto S, Supari S. Rainfall Changes over Java Island, Indonesia. J Environ Earth Sci. 2015;5: 187-195.

33. Paredes Trejo FJ, Barbosa HA, Peñaloza-Murillo MA, Alejandra Moreno M, Farías A. Intercomparison of improved satellite rainfall estimation with CHIRPS gridded product and rain gauge data over Venezuela. Atmosfera. 2016;29.

34. Nawaz M, Iqbal MF, Mahmood I. Validation of CHIRPS satellite-based precipitation dataset over Pakistan. Atmos Res. 2021;249.

35. Shen Z, Yong B, Gourley JJ, Qi W, Lu D, Liu J, et al. Recent global performance of the Climate Hazards group Infrared Precipitation (CHIRP) with Stations (CHIRPS). J Hydrol. 2020;591.

36. Liu Z, Ostrenga D, Teng W, Kempler S. Tropical rainfall measuring mission (TRMM) precipitation data and services for research and applications. Bull Am Meteorol Soc. 2012;93: 1317-1325.

37. Kubota T, Shige S, Hashizume H, Aonashi K, Takahashi N, Seto S, et al. Global precipitation map using satellite-borne microwave radiometers by the GSMaP project: Production and validation. In: IEEE Transactions on Geoscience and Remote Sensing. 2007.

38. Sorooshian S, Hsu KL, Gao X, Gupta H V, Imam B, Braithwaite D. Evaluation of PERSIANN system satellite-based estimates of tropical rainfall. Bull Am Meteorol Soc. 2000;81: 2035-2047.

39. Nguyen P, Ombadi M, Sorooshian S, Hsu K, AghaKouchak A, Braithwaite D, et al. The PERSIANN family of global satellite precipitation data: A review and evaluation of products. Hydrol Earth Syst Sci. 2018;22: 5801-5816.

40. Nguyen P, Shearer EJ, Tran H, Ombadi M, Hayatbini N, Palacios T, et al. The CHRS data portal, an easily accessible public repository for PERSIANN global satellite precipitation data. Sci Data. 2019;6: 180296.

41. van den Besselaar EJM, van der Schrier G, Cornes RC, Suwondo A, Iqbal, Tank AMGK. SA-OBS: A daily gridded surface temperature and precipitation dataset for Southeast Asia. J Clim. 2017;30: 5151-5165.

42. Yatagai A, Kamiguchi K, Arakawa O, Hamada A, Yasutomi N, Kitoh A. Aphrodite constructing a long-term daily gridded precipitation dataset for Asia based on a dense network of rain gauges. Bull Am Meteorol Soc. 2012;93: 1401-1415.

43. Yasutomi N, Hamada A, Yatagai A. Development of a long-term daily gridded temperature dataset and its application to rain / snow discrimination of daily precipitation. Glob Environ Res. 2011;15.

44. Joyce, R. J., Janowiak, J. E., Arkin, P. A., & Xie, P. (2004). CMORPH: A Method that Produces Global Precipitation Estimates from Passive Microwave and Infrared Data at High Spatial and Temporal Resolution, Journal of Hydrometeorology, 5(3), 497-513

45. Huffman GJ, Adler RF, Bolvin DT, Gu G, Nelkin EJ, Bowman KP, et al. The TRMM Multisatellite Precipitation Analysis (TMPA): Quasi-global, multiyear, combined-sensor precipitation estimates at fine scales. J Hydrometeor. 2007;8.

46. Song Z, Xia J, She D, Zhang L, Hu C, Zhao L. The development of a Nonstationary Standardized Precipitation Index using climate covariates: A case study in the middle and lower reaches of Yangtze River Basin, China. J Hydrol. 2020;588.

47. Mishra AK, Singh VP. A review of drought concepts. J Hydrol. 2010;391: 202-216.

48. Hsin-Fu Yeh HF, Chang CF. Using standardized groundwater index and standardized precipitation index to assess drought characteristics of the Kaoping River Basin, Taiwan. Water Resour. 2019;47: 670-678.

49. Lloyd-Hughes B, Saunders MA. A drought climatology for Europe. Int J Climatol. 2002;22: 1571-1592.

50. WMO. Standardized precipitation index user guide. J Appl Bacteriol. 2012;63: 197-200.

51. Adams. Climate Indices for Drought Monitoring, Community Reference Implementations in Python [Internet]. 2017. Available from: https://github.com/monocongo/climate_indices.

Fig1

Click here to access/download;Figure;Fig1.tif

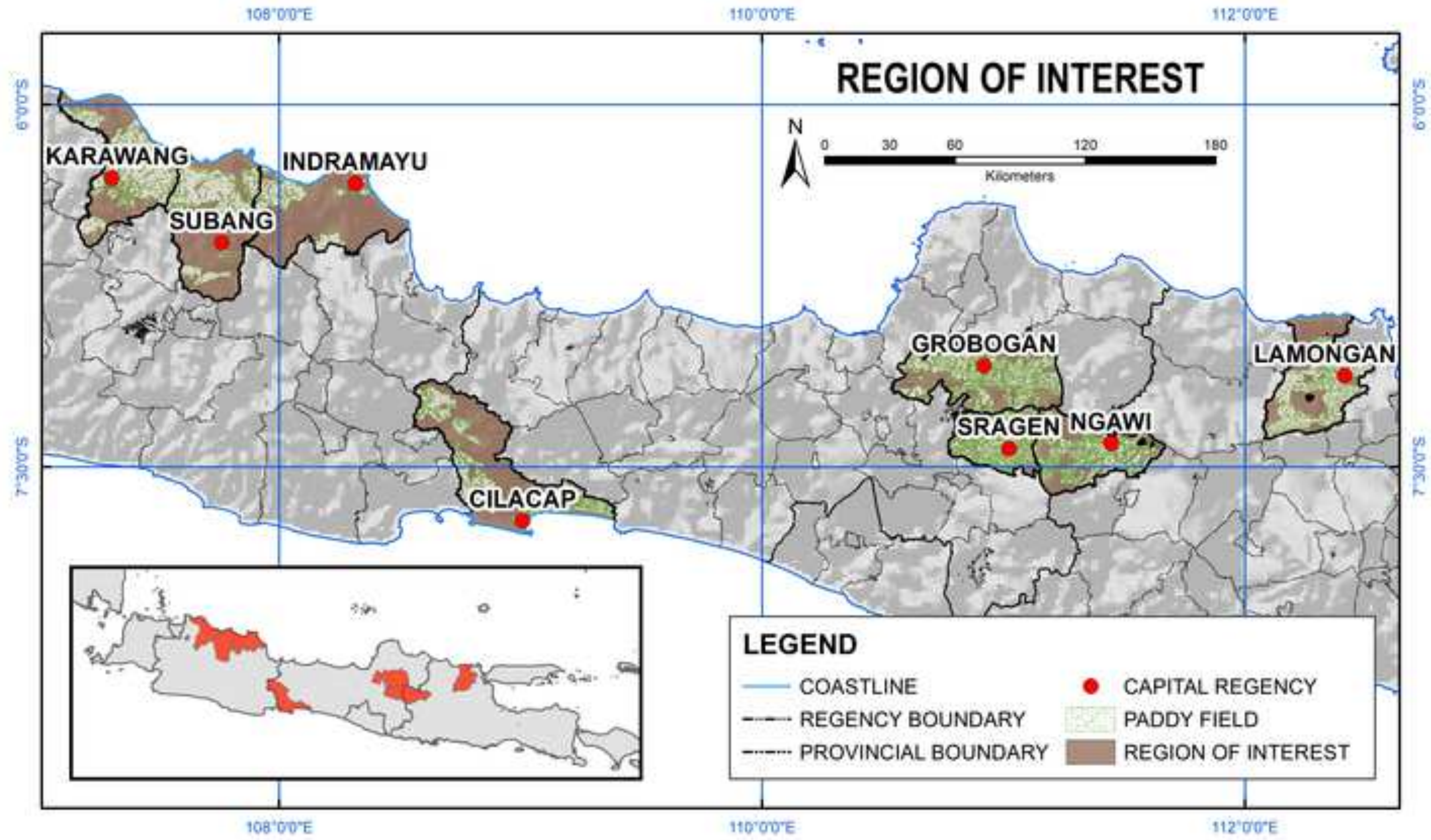

Fig2

Click here to access/download;Figure;Fig2.tif

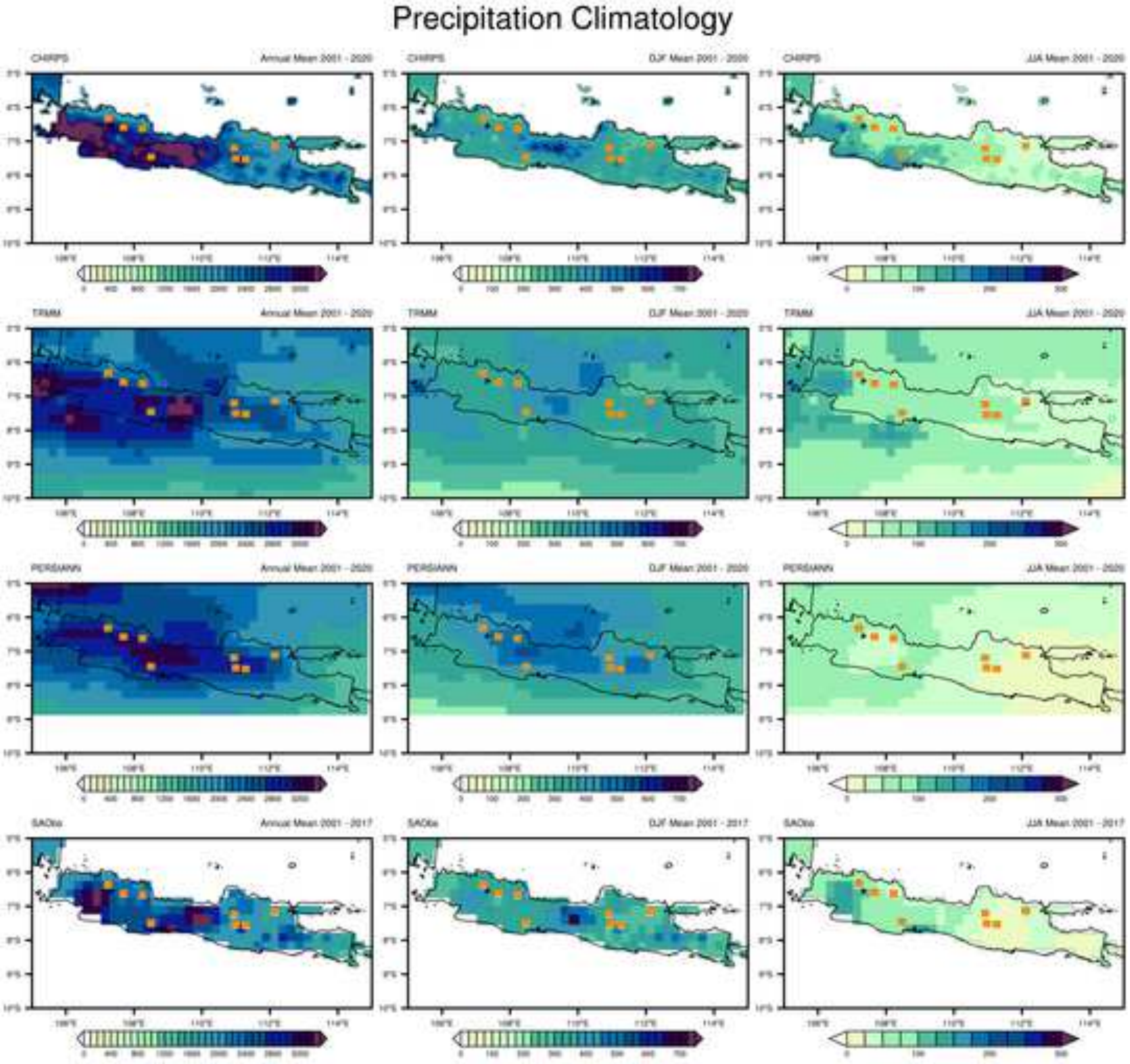

Fig3

Click here to access/download;Figure;Fig3.tif

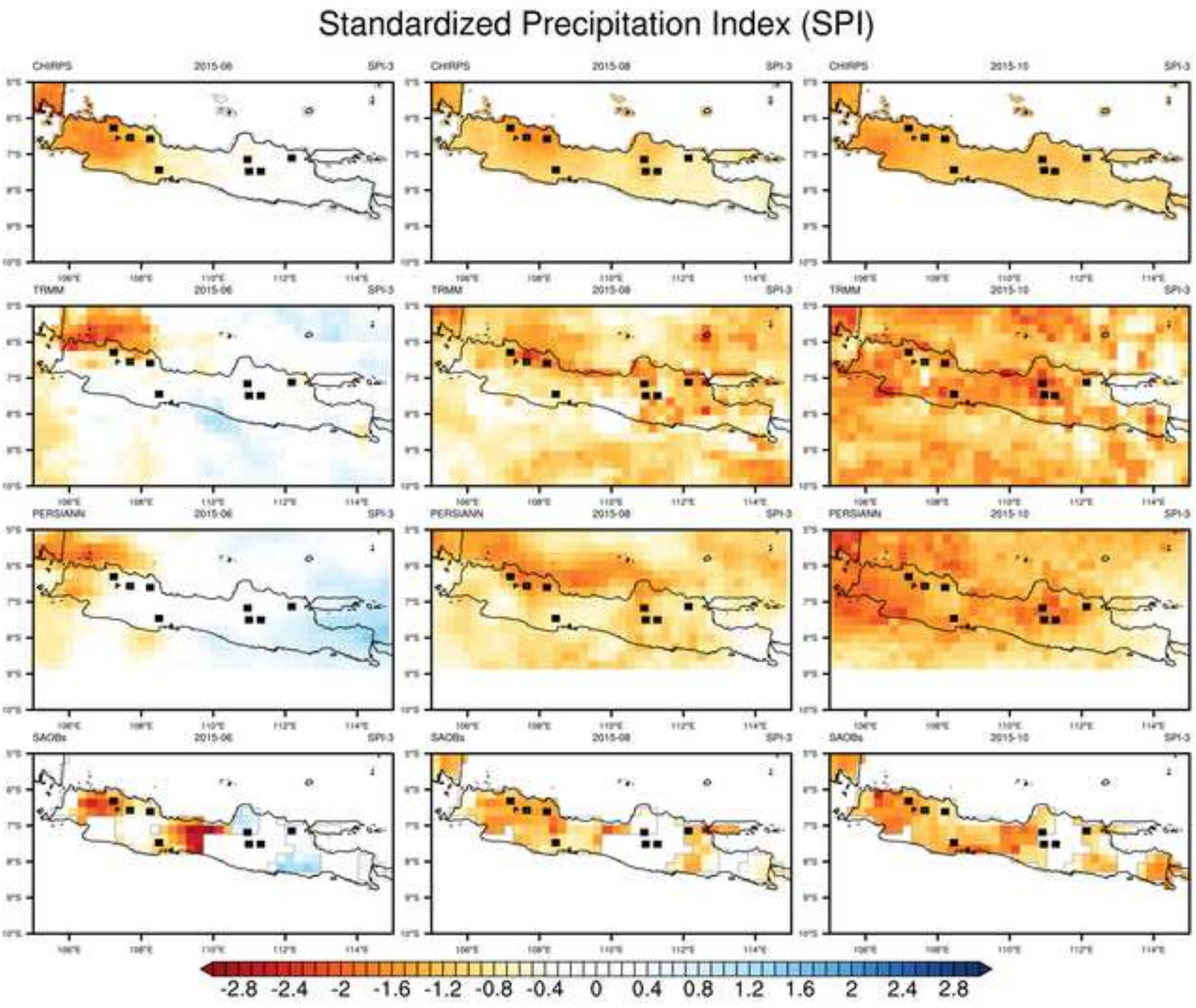

Fig4

Click here to access/download;Figure;Fig4.tif

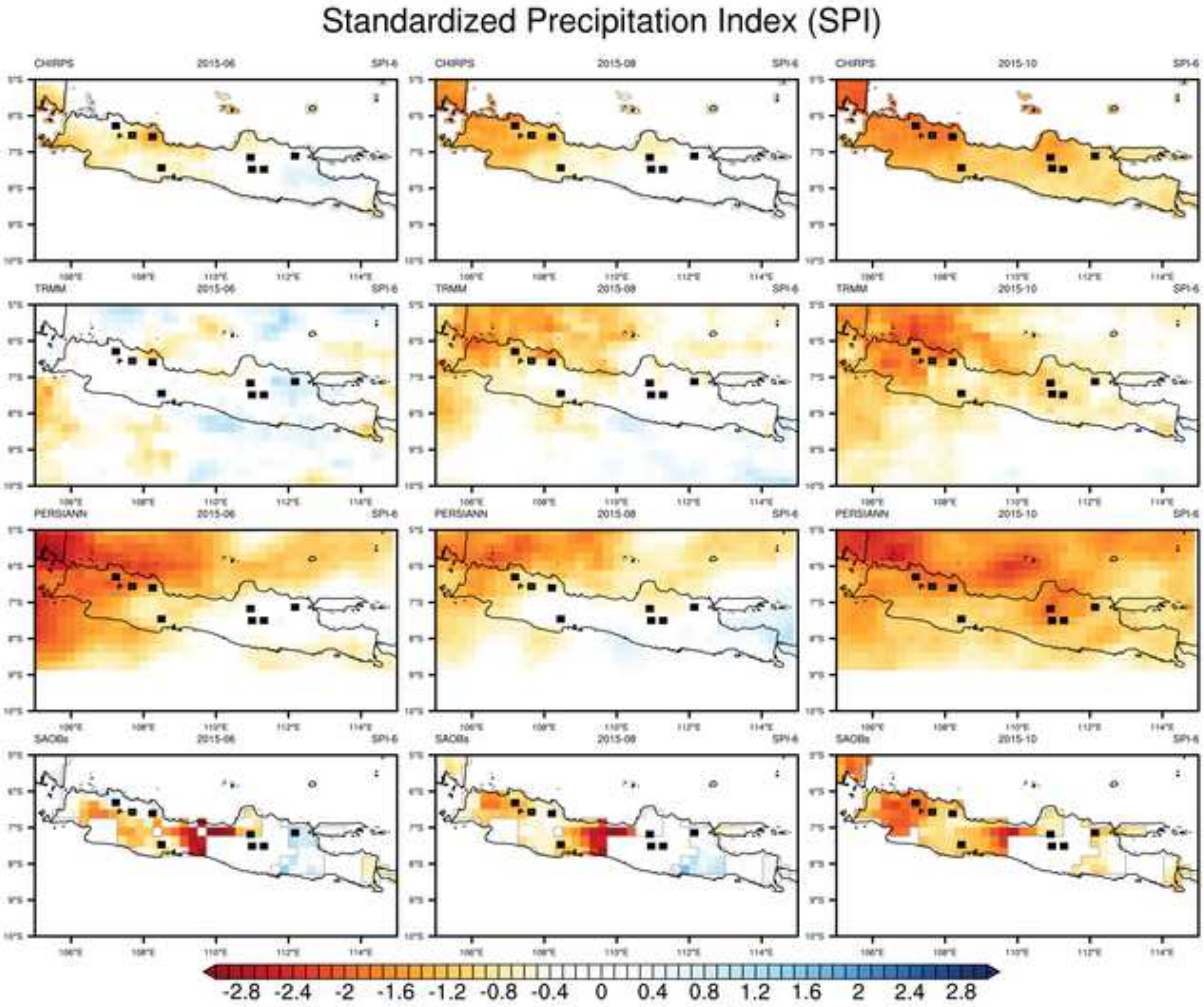

Fig5 Click here to access/download;Figure;Fig5.tif

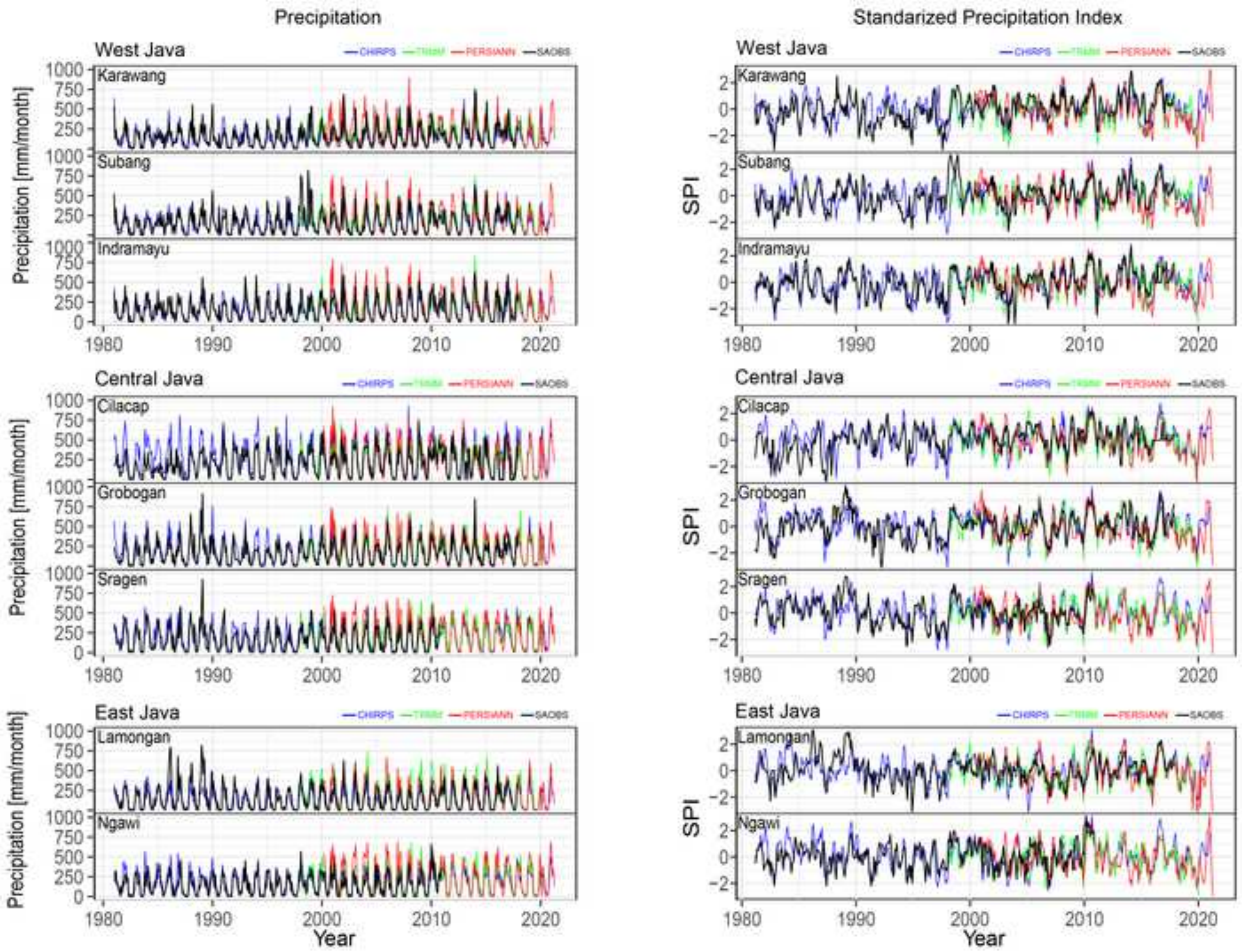

Fig6

Click here to access/download;Figure;Fig6.tif

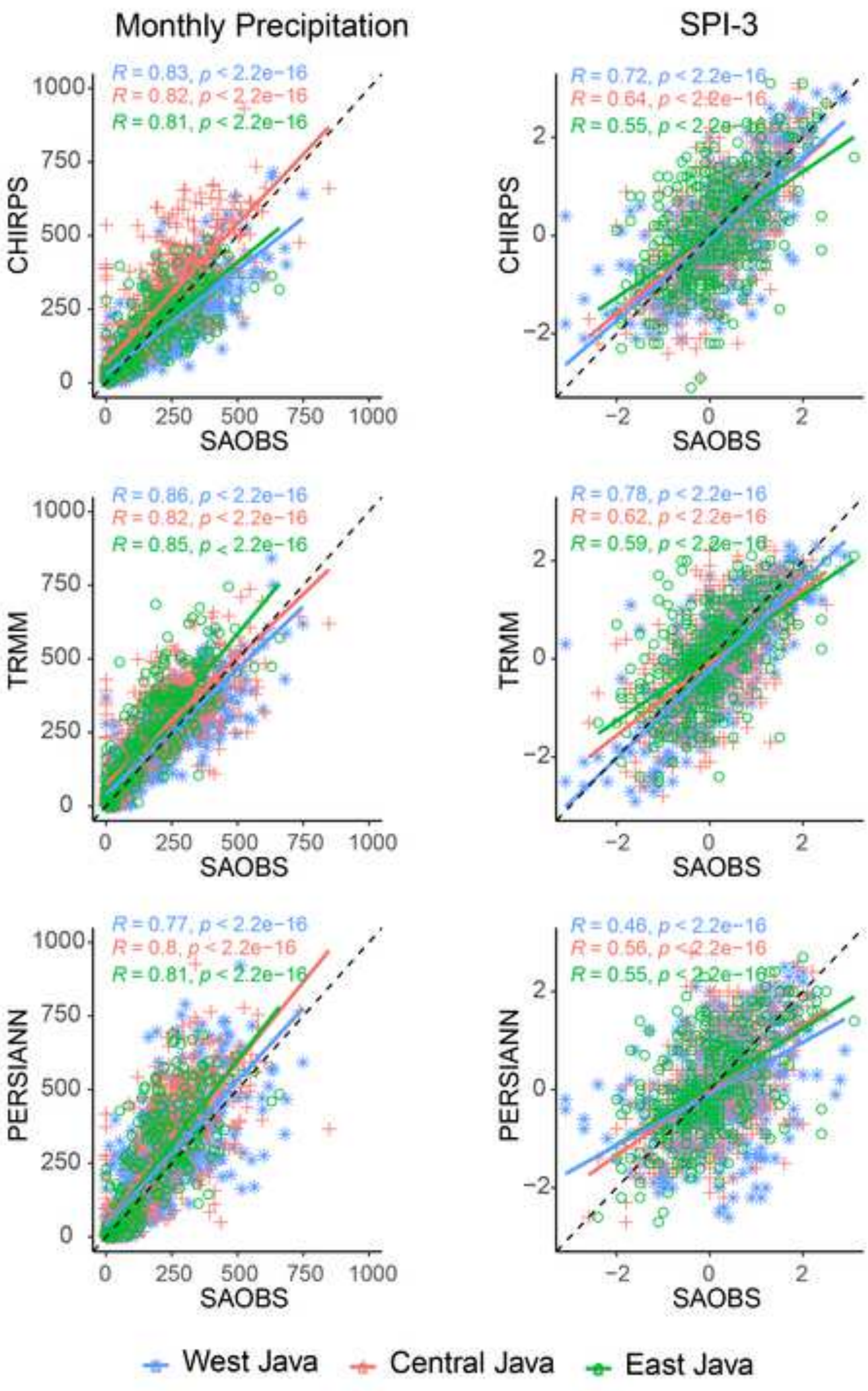

Fig7

Click here to access/download;Figure;Fig7.tif

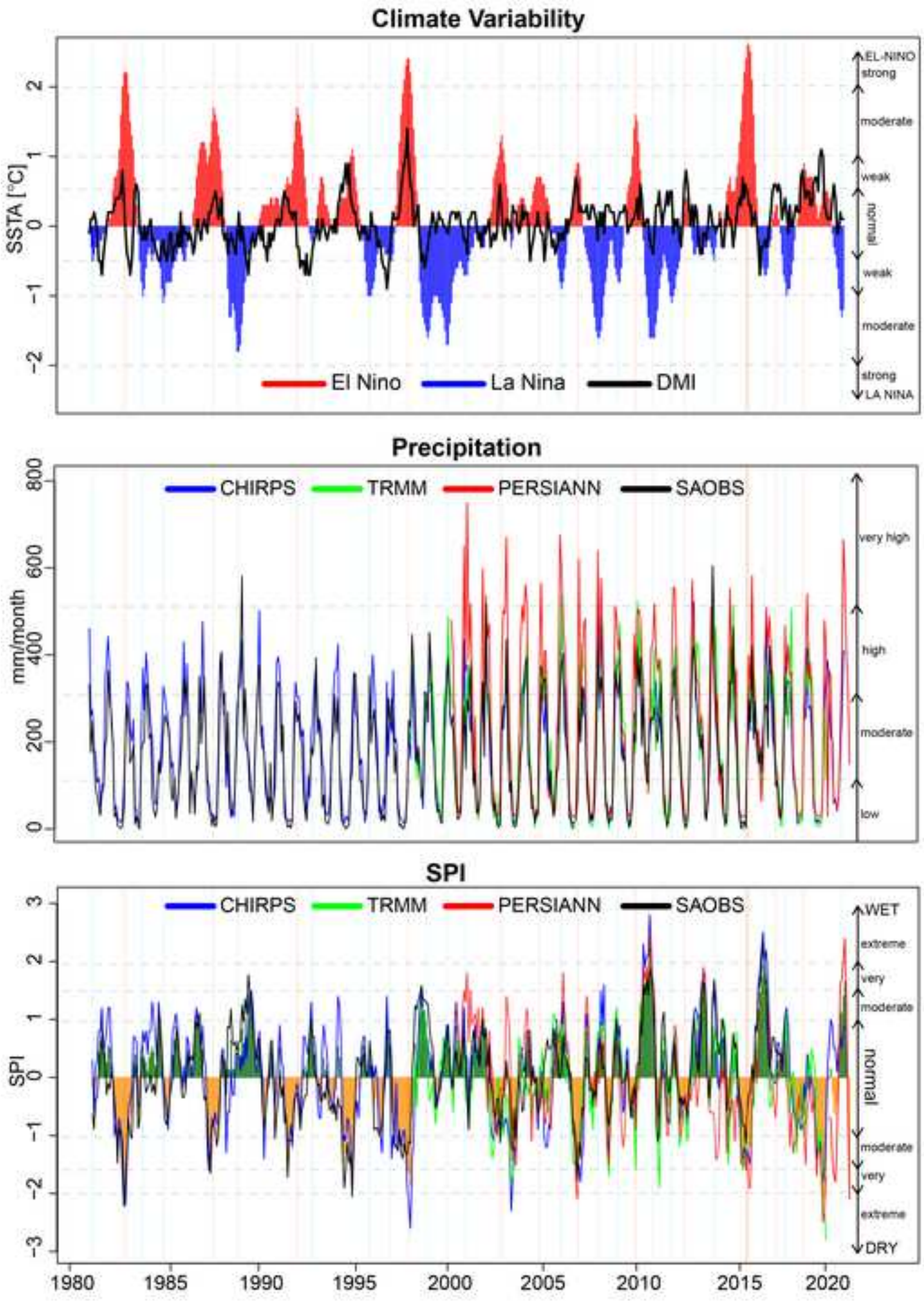

Click here to access/download
Supporting Information
Supporting Infromation.docx
