# Supplementary material for: Satellite-based meteorological drought indicator to support food security in Java Island
Source: PLoS One. 2022 Jun 3;17(6):e0260982. doi: 10.1371/journal.pone.0260982 (PMC9165873; doi:10.1371/journal.pone.0260982)
Supplement: Attachment — Submitted filename: Respon to Reviewer #2.docx [file pone.0260982.s003.docx]

**Reply to the Reviewer #2**

We would like to thank the editor and reviewer#2 for his/her valuable comments. We have revised the manuscript and we hope that all comments and suggestions have been addressed and considered accordingly.

Abstract

1. How did you developed meteorological drought?

- we revised our mistaken previous statements :

“a meteorological drought indicator was obtained by applying the Standardized Precipitation Index (SPI) to satellite-based precipitation products from multiple sources. The SPI based meteorological drought analysis was then applied to Java Island, in particular to the largest rice-producing districts of Indonesia.”

2. Too many keywords, six keywords should suffice and rearrange the keywords alphabetically

- we reduced the number of keywords: Meteorological drought, Satellite- based precipitation, SPI, Java Island, El-Nino

Introduction

1. More citation should be included

- we have added more citations in the Introduction and Discussion.

- we apologise to not using the list papers as suggested by the reviewers, since we believed that those mentioned papers more suitable in the discussion of agricultural drought, which is out of the scope of this article.

2. What is the identified gap that this study is filling? This is not clearly stated, address this during revision

- we revised the filling gap part in the text, hope it can be sufficient:

“This differs from currently available where the SPI is calculated based on sparse surface observation data. The evaluation of multi-source SPI with El Niño and IOD simultaneously is expected to provide a better, optimal, and more accurate representation and understanding of meteorological drought events in the study area. We used the SPI based meteorological drought indicator to evaluate the satellite precipitation products against ground observation data for Java Island, with a focus on eight of the 10 largest rice-producing districts of Indonesia”

Result and discussion

1. You failed to compare your result with recent and televant studies.

- we are now adding more citations in the discussion from very recently relevant studies. Hope now the Discussion section is becoming better.

Conclusion

1. No citation is expected in the conclusion

- We deleted the citation, thanks.
